# Neuronal Death in the Contralateral Un-Injured Retina after Unilateral Axotomy: Role of Microglial Cells

**DOI:** 10.3390/ijms20225733

**Published:** 2019-11-15

**Authors:** Fernando Lucas-Ruiz, Caridad Galindo-Romero, Kristy T. Rodríguez-Ramírez, Manuel Vidal-Sanz, Marta Agudo-Barriuso

**Affiliations:** 1 Group de Oftalmología Experimental, Instituto Murciano de Investigación Biosanitaria Virgen de la Arrixaca (IMIB-Arrixaca), 30120 Murcia, Spain; fer138lucas@gmail.com (F.L.-R.); caridad.galindo@um.es (C.G.-R.); kristytatiana.rodriguez@um.es (K.T.R.-R.); manuel.vidal@um.es (M.V.-S.); 2 Departamento de Oftalmología, Facultad de Medicina, Universidad de Murcia, 30100 Murcia, Spain

**Keywords:** optic nerve crush, retinal ganglion cells (RGC), meloxicam, minocycline, bilateral response, visual system

## Abstract

For years it has been known that unilateral optic nerve lesions induce a bilateral response that causes an inflammatory and microglial response in the contralateral un-injured retinas. Whether this contralateral response involves retinal ganglion cell (RGC) loss is still unknown. We have analyzed the population of RGCs and the expression of several genes in both retinas of pigmented mice after a unilateral axotomy performed close to the optic nerve head (0.5 mm), or the furthest away that the optic nerve can be accessed intraorbitally in mice (2 mm). In both retinas, RGC-specific genes were down-regulated, whereas caspase 3 was up-regulated. In the contralateral retinas, there was a significant loss of 15% of RGCs that did not progress further and that occurred earlier when the axotomy was performed at 2 mm, that is, closer to the contralateral retina. Finally, the systemic treatment with minocycline, a tetracycline antibiotic that selectively inhibits microglial cells, or with meloxicam, a non-steroidal anti-inflammatory drug, rescued RGCs in the contralateral but not in the injured retina. In conclusion, a unilateral optic nerve axotomy triggers a bilateral response that kills RGCs in the un-injured retina, a death that is controlled by anti-inflammatory and anti-microglial treatments. Thus, contralateral retinas should not be used as controls.

## 1. Introduction

Optic nerve axotomy is a great model to study in vivo the effect of a localized axonal trauma to the central nervous system (CNS). In the rodent retina, this lesion specifically affects retinal ganglion cells (RGCs) without causing the retrograde death of other retinal neurons, [1] although displaced amacrine cells in the ganglion cell layer exhibit some nuclear atrophy [2].

The course of RGC death induced by optic nerve axotomy has been thoroughly studied by our group and others [3,4,5,6,7,8,9,10,11]. In the injured retina, RGC loss is apoptotic [6] and occurs in two phases. During the first 9–14 days, 85% of RGCs are lost. Thereafter, RGCs die more slowly, and by day 90 only 1.5% survive (reviewed in [12]).

In rats, it has been shown that the closer to the optic head the axotomy is inflicted, the quicker the death of RGCs [9]. This is observed when comparing the course of RGC loss between two intraorbital distances or between intraorbital and intracranial axotomies. This effect has not been yet been investigated in mice.

Since the late 1990s, it is known that the impact of an unilateral optic nerve injury is not confined to the injured retina [13]. Since then, this observation has been corroborated and the knowledge of the bilateral response broadened in different animal models and in humans [14,15,16,17,18,19,20,21], which has been reviewed in [22]. For instance, in healthy humans it has been shown that when the perfusion of one eye is reduced experimentally there is a bilateral decrease of the retinal function [17]. In mice, Lam et al. [19] showed that the insertion of an iron wire into one eye triggers a bilateral increase of free radicals and of low molecular weight polypeptides, responses that are controlled by cyclosporine (immunosuppressant) treatment. Unilateral ocular hypertension or optic nerve axotomy (crush or transection) in rodents cause macrogliosis, microglial activation, division and phagocytosis, up-regulation of the major histocompatibility complex class II (MHCII), and aberrant expression of the highly phosphorylated axonal neurofilament subunit H [14,15,16,18,20,21,22] in the un-injured retina. Interestingly, in albino rats, Macharadze et al. [14] observed, besides microglial activation, that there was loss of ipsilateral RGCs in the un-injured retina, a loss that was controlled by dexamethasone. Importantly, this bilateral response to a unilateral stimuli is not elicited only after harmful stimuli but also after apparently innocuous treatments, such as topical administration of brimonidine that causes the up-regulation of growth factors in both retinas [21].

In pigmented mice, we reported that the number of RGCs in the contralateral retinas after unilateral optic nerve crush (ONC) performed at 0.5 mm from the optic head was lower than in intact ones at 14 days, although this decrease did not reach statistical significance [4]. We also observed the presence of phagocytic microglial cells in the contralateral retina, a response that was constant at least up to 14 days, and it was not modulated by the rate of RGC loss in the injured retina. The presence in the contralateral retinas of phagocytic microglial cells filled with fluorogold from traced-RGCs, together with the fact that the number of RGCs in these retinas decreased, led us to think that a unilateral axotomy could induce RGC death in the contralateral retina that may reach significance a longer times post-lesion.

Hence, in this work, we had three complementary objectives. Firstly, to assess whether RGC death in mice is modulated by the distance at which the intraorbital axotomy is performed; secondly, to find out if there is RGC loss in the contralateral un-injured retinas of these animals; and finally, to investigate whether the contralateral loss of RGCs is controlled by systemic anti-inflammatory or anti-microglial treatments.

The optic nerve of adult pigmented mice was crushed at 0.5 mm or at 2 mm from the optic disk. It is important to highlight here that the distance to the lesion site inverts for the contralateral retinas, and so ONC 2 mm is closer and ONC 0.5 mm is farther. Our results showed that in the injured retinas, the course of RGC loss did not differ between lesion sites. In the contralateral retinas, there was a fixed loss of 15% of RGCs in both groups that was first identified after the ONC 2 mm. Finally, we showed that the direct microglial inhibition with minocycline, [23,24,25,26] or the treatment with meloxicam, a non-steroidal anti-inflammatory drug (NSAID, [27,28]), rescued all RGCs in the contralateral un-injured retina, but did not have an effect on the injured one.

## 2. Results

### 2.1. The Course of RGC Death in the Injured Retinas is not Affected by the Intraorbital Distance

**Figure 1:**
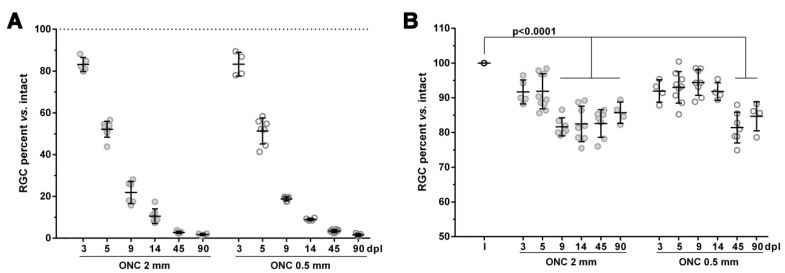
Retinal ganglion cell loss in injured and contralateral retinas: influence of the lesion distance. Scatter graphs showing the percent versus intact (100%, dotted line in (**A**); I in (**B**)) of surviving retinal ganglion cells (RGCs). Black lines indicate the mean percent ± standard deviation. (**A**) In the injured retinas and in both experimental paradigms, RGC death was first significant at day 3, progressed steeply up to 14 days, and slowly thereafter. There were no significant differences between optic nerve crush (ONC) 0.5 mm and ONC 2 mm at any time point. (**B**) In the contralateral retinas, there was a significant decrease of RGCs at day 9 in the ONC 2 mm group, which did not progress further. In the ONC 0.5 mm group, RGC loss reached statistical significance at 45 days, and it also did not progress at 90 days. Statistics: one-way ANOVA, Kruskal–Wallis, Dunn′s multiple comparisons tests. See Table 1 for more details. dpl: days post-lesion.

**Figure 2:**
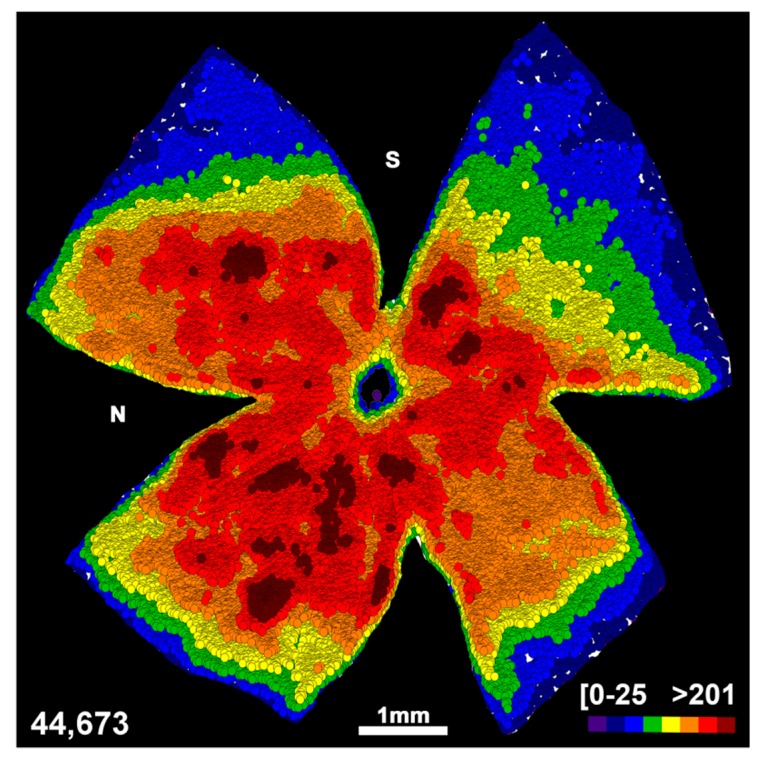
Retinal ganglion cell topography in intact retinas. Neighbour map illustrating the topography of RGCs in a representative intact retina. Neighbour maps illustrate the number of RGCs around a given RGC in a radius of 0.200 mm with a colour scale (bottom right) from 0–25 neighbours (purple) to >201 neighbours (dark red). The bottom left of the map shows the number of RGCs counted in the original retina. S: superior; N: nasal.

**Figure 3:**
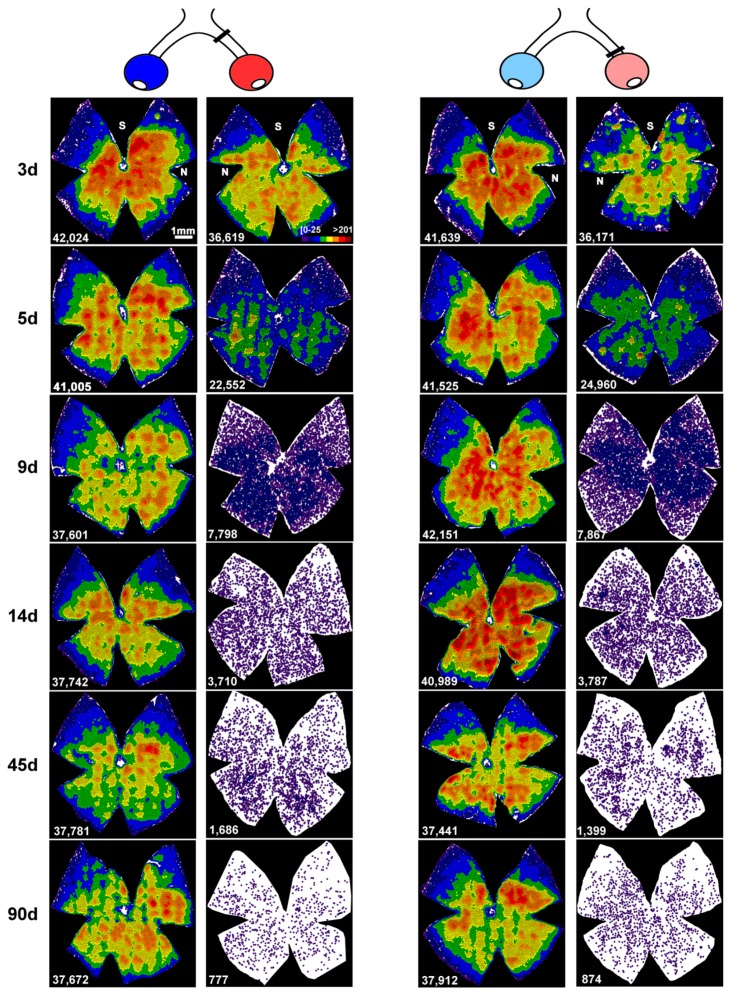
Topography of retinal ganglion cell loss in the injured and contralateral retinas. Top, drawings representing both eyes, their optic nerves, and the lesion site. Left: ONC performed at 2 mm. Right: ONC performed at 0.5 mm. Blue eyes: contralateral. Red: injured. Below: neighbour maps illustrating the topography of surviving RGCs from 3 to 90 days in representative retinas from each group. These neighbour maps illustrate the number of RGCs around a given RGC in a radius of 0.200 mm with a colour scale (top, second from the left panel) from 0–25 neighbours (purple) to >201 neighbours (dark red). The bottom left of each map shows the number of RGCs counted in the original retina. d: days post-lesion; S: superior; N: nasal.

First, we compared the course of RGC death between retinas axotomized at 0.5 mm and at 2 mm from the optic disk. On the basis of previous studies on rats [1,9], we hypothesised that in mice the death of RGCs would also be quicker when the ONC was performed closer to the retina. To our surprise, the loss of RGCs was the same quantitatively and topographically, irrespective of the lesion site (Figure 1, Figure 2 and Figure 3, Table 1). In agreement with previous reports [3,6], RGC death was first significant at day 3 after the ONC and proceeded quickly up to 14 days, when ~9% of the original population of RGCs survived. Thereafter, RGC death continued but at a much slower rate, and by day 90 only 1.5% of RGCs were still present.

### 2.2. An Unilateral Axotomy Causes RGC Loss in the Contralateral Un-Injured Retina Whose Course Is Influenced by the Lesion Distance

When we analyzed the contralateral un-injured retinas, we observed in both groups a non-significant decrease of 7–9 ± 1.5% of RGCs already at 3 days (Figure 1, Figure 2 and Figure 3 and Table 1). This loss reached significance at 9 days in the ONC 2 mm group and at 45 days in the ONC 0.5 mm (mean loss of 18 ± 2% of the original population in both groups) and did not proceed further, at least up to 90 days (15 ± 3% decrease at this time point in both groups). Of note, in the ONC 0.5 mm, the first significant time point of RGC loss may have been earlier than at 45 days, at any time between 14 and 45 days. Topographically, RGCs died all across the retina, as evidenced by the loss of warm colours (higher number of RGCs in a given location) in the neighbour maps (compare the distribution of RGCs between intact and contralateral retinas in Figure 2 and Figure 3).

### 2.3. Transient Down-Regulation of Brn3a Expression in the Surviving RGCs

Since we characterized Brn3a as a marker of RGCs [5], we have been noticing that in the injured retinas the signal intensity of Brn3a immunodetection was always weaker than in intact retinas. In accordance with this observation, we reported that in rats the expression of Brn3a in the injured retinas decreased for two reasons: (i) the death of RGCs, and (ii) a down-regulation of this protein in the surviving ones [29]. Moreover, Brn3a down-regulation signals apoptotic RGC death [6] and correlates with the course of RGC loss, that is, the quicker the model of RGC death, the higher the down-regulation of Brn3a per RGC [29].

**Figure 4:**
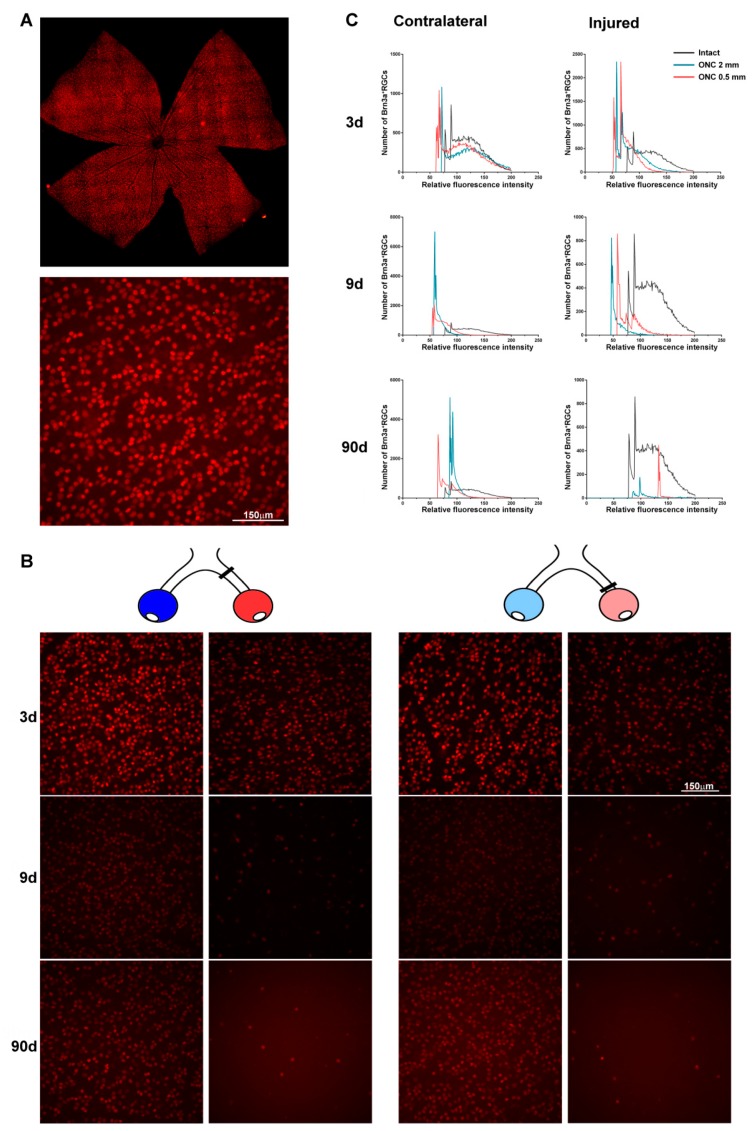
Brn3a expression in the injured and contralateral retinas: RGC death and transient down-regulation. (**A**) Brn3a immunodetection in a retinal photomontage (top) from the intact group, and a magnified view (bottom). (**B**) Brn3a magnifications from injured and contralateral retinas analysed at 3, 9, and 90 days after ONC 2 mm and ONC 0.5 mm. These images and the magnification in (**A**) were acquired with the same settings. In the injured and contralateral retinas, Brn3a signal was less bright than in intact retinas. At 90 days post-lesion, the signal of Brn3a per nuclei was stronger than at earlier time points. d: days post-lesion. (**C**) *x-, y*-plot showing the distribution of RGCs according to their expression level of Brn3a. The number of RGCs (ordinate axis) in intact retinas, injured and contralateral retinas analysed 3, 9, or 90 days after both ONC lesions was plotted against the intensity of their Brn3a signal. In both retinas, the mean intensity of Brn3a expression per nucleus decreased compared to intact retinas at 3 and 9 days but recovered at 90 days.

The same phenomenon occurs in mice and it was observed in both retinas, the injured and the contralateral. Furthermore, at longer time post-lesions the surviving RGCs seemed to have a brighter Brn3a signal (Figure 4). Measurement of the level of Brn3a signal intensity per nuclei confirmed this observation—the relative fluorescence level per RGC shifted to the left (lower intensity) at 3 and 9 days, a shift that was bigger in the injured than in the contralateral retinas. At 90 days, Brn3a signal per cell fell within intact values.

### 2.4. mRNA Regulation of RGC Markers in the Injured and Contralateral Retinas

Due to the transient down-regulation of Brn3a observed by immunohistofluorescence, we wondered whether this was a common feature of RGC-specific genes. Thus, we measured the expression level of three known RGC-specific transcripts, *Pou4f1* (protein Brn3a [5]), *Rbpms* (protein RNA-binding protein with multiple splicing [30]), and *Sncg* (protein γ-synuclein, [31]). These transcripts encode proteins that in the adult mice retina are only expressed by RGCs, and therefore their levels reflect the state of this neuronal population.

**Figure 5:**
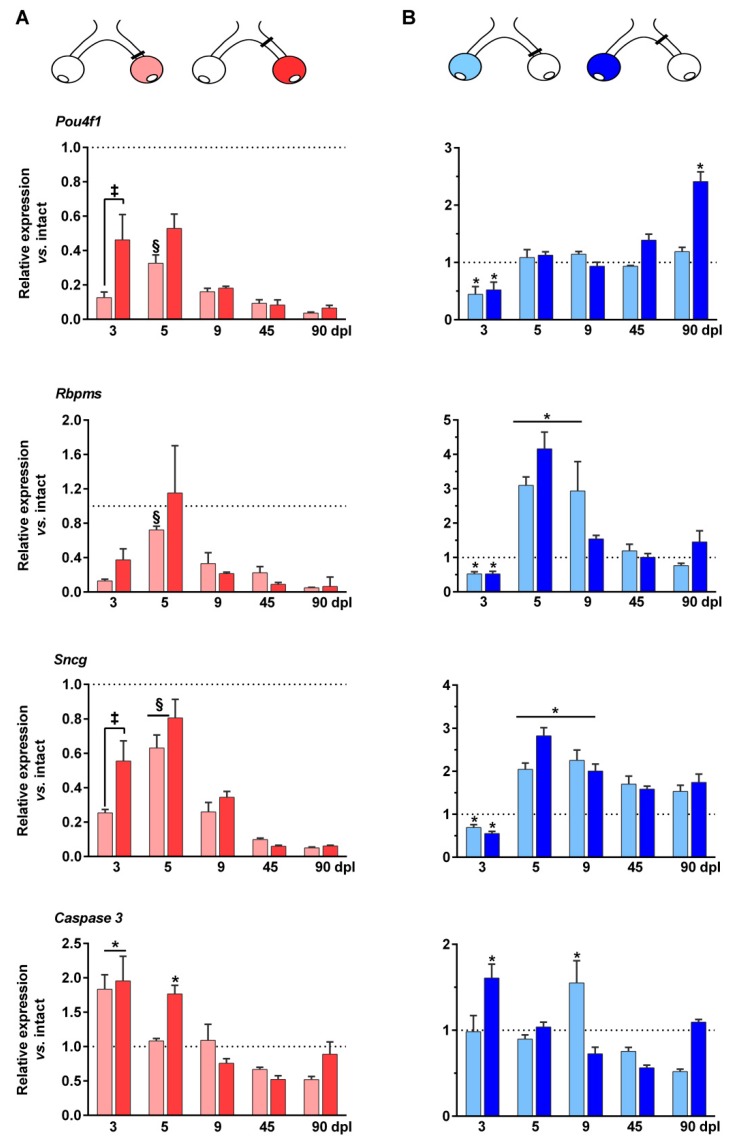
Expression levels of RGC markers and caspase 3 in the contralateral and injured retinas. Bar graphs showing the expression level ± S.E.M. of *Pou4f1* (Brn3a), *Rbpms* (protein RNA-binding protein with multiple splicing)*, Sncg* (γ-synuclein) and *Caspase 3*, transcripts in the injured ((**A**) red) and contralateral ((**B**) blue) retinas at increasing time points after ONC 0.5 mm (light red or blue) and ONC 2 mm (dark red or blue) compared to intact retinas (value 1, dotted lines). In the injured retinas, the expression of *Pou4f1, Rbpms*, and *Sncg* was significantly lower than in intact retinas at all time points and in both groups except for *Rbmps* at 5 days post-lesion and *Sncg* at 5 days in the ONC 2 mm group. * Significant compared to intact retinas. ^§^ Significant compared to the previous time point, ^‡^ Significant between ONC 0.5 and ONC 2 mm (*p* < 0.05, ANOVA).

As expected, in the injured retinas all of these markers were significantly down-regulated at day 3 post-lesion and from 9 days onwards their levels decreased steadily (Figure 5A). Interestingly, at day 3 *Pou4*f1 and *Sncg* transcript levels were significantly higher after ONC 2 mm than after ONC 0.5 mm. *Rbpms* regulation at this time point showed the same trend, but did not reach significance. This may indicate that although the loss of RGCs does not differ between injuries, ONC 2 mm causes a slightly slower molecular decline. Unexpectedly, at day 5 the expression of the three mRNAs was up-regulated compared to day 3 (*Pou4f1* and *Rbpms* after ONC 0.5 mm, and *Sncg* in both lesions). It is possible that this transient up-regulation is an attempt of RGCs to overcome the injury.

In the contralateral un-injured retina (Figure 5B) something similar occurred—at day 3 the three markers were down-regulated, and then they recovered either to normal levels (*Pou4f1*) or above normal levels (*Rbpms* and *Sncg*). *Sncg* was up-regulated up to 90 days, whereas *Rbmps* came back to normal at 45 days. *Pou4f1* regulation was different- after ONC 2 mm its levels raised at 45 days, and at 90 days it was significantly up-regulated compared to intact retinas, in accordance with the immunofluorescence signal of Brn3a. In the ONC 0.5 mm lesion, this up-regulation was not observed, probably because in this paradigm RGC loss in the un-injured retinas was observed later and, thus, it is possible that longer time post-lesions are necessary to see this phenomenon.

All in all, these data suggest that RGCs in the injured and contralateral retinas responded similarly, down-regulating gene expression early after the lesion followed by a wave of up-regulation in the surviving RGCs.

### 2.5. Up-Regulation of Caspase 3 mRNA Expression in the Injured and Contralateral Retinas

Optic nerve axotomy causes the death of RGCs by apoptosis, at least during the first quick phase of RGC loss (up to 9 days [6]). We have shown that in retinas axotomized at 0.5 mm the number of RGCs expressing the cleaved form of caspase 3 peaks at day 4 [6]. Here, we measured caspase 3 mRNA expression level and found that in the injured retinas caspase 3 mRNA was over-expressed at day 3 after ONC 0.5 mm and at 3 and 5 days after ONC 2 mm (Figure 5A). As observed for *Pou4f1* and *Sncg*, this may indicate that although anatomically the loss of RGCs is the same for both lesions, molecularly the signals may be slightly delayed in the ONC 2 mm group.

In the contralateral retinas, caspase 3 over-expression was observed at day 3 and 9 in the ONC 2 mm and 0.5 mm groups, respectively (Figure 5B). This concords with the fact that when the lesion was performed at 2 mm, the loss of RGCs in the contralateral retina was observed earlier than after ONC 0.5 mm.

### 2.6. Treatment with Minocycline or Meloxicam Rescues RGCs in the Contralateral Retinas

In the contralateral retinas there is a significant activation of microglial cells [4,13,16] and an inflammatory response [32]. Thus, we hypothesised that the direct inhibition of microglial cells with minocycline or dampening the inflammatory response with an NSAID (meloxicam) could be beneficial.

**Figure 6:**
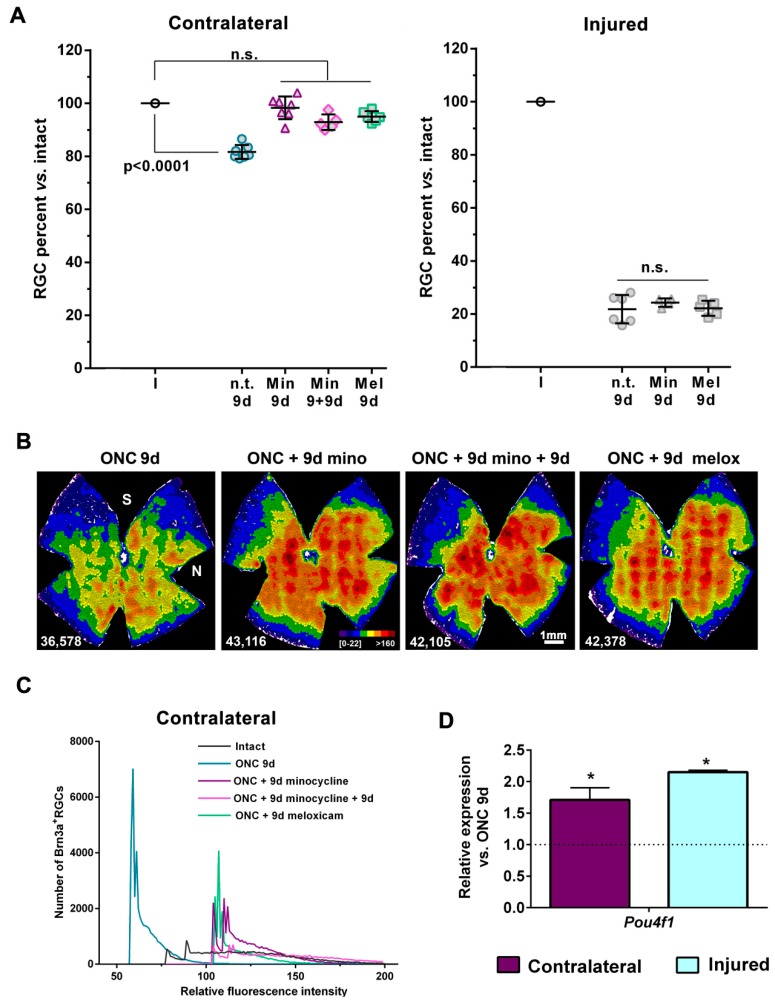
Retinal ganglion cell death in the contralateral retina was rescued by microglial inhibition and by NSAID treatment. (**A**) Scatter graphs showing the percent versus intact (I, 100%) of RGCs in contralateral and injured retinas from animals treated with minocycline (Mi), meloxicam (Mel), or non-treated (n.t.). Minocycline and meloxicam treatments for 9 days rescued RGCs in the contralateral retinas, and this effect lasted 9 days after the last minocycline injection (Mi + 9d). In the injured retinas, however, microglial inhibition did not rescue RGCs. Statistics: one-way ANOVA, Kruskal–Wallis, Dunn′s multiple comparisons tests. n.s.: non-significant. See Table 1 for more details. (**B**) Neighbour maps illustrating the topography of surviving RGCs in contralateral retinas from the same groups as in (**A**). These neighbour maps illustrate the number of RGCs around a given RGC in a radius of 0.200 mm with a colour scale (second from the left panel) from 0–25 neighbours (purple) to >160 neighbours (dark red). The bottom left of each map shows the number of RGCs counted in the original retina. S: superior, N: nasal. (**C**) Minocycline treatment maintained Brn3a expression per RGC in the contralateral retinas. *x-, y*-plot showing the distribution of RGCs according to their expression level of Brn3a. The number of RGCs (ordinate axis) from the groups in (**A**), is plotted against the intensity of their Brn3a signal (*y-*axis). The mean intensity of Brn3a expression per nucleus in the contralateral treated retinas was within intact levels. (**D**) Minocycline treatment increased Brn3a mRNA expression in injured and contralateral retinas compared to untreated ones. Bar graphs showing the expression level ± S.E.M. of *Pou4f1* (Brn3a transcript) in the injured and contralateral retinas from the ONC + minocycline 9 day group compared to ONC 9 days (dotted line, value 1). * *p* < 0.05 compared to ONC 9 days *(t-*test). *n* = 4 retinas per group.

We chose 9 days after ONC 2 mm because this was when RGC death in the contralateral retinas was first significant. As shown in Figure 6A,B and Table 1, daily treatment with minocycline or meloxicam rescued all RGCs in the contralateral retina, but it did not have an effect on the injured retina (Figure 6A, Table 1), as expected [33]. Of note, in non-lesioned animals these same treatments did not have a negative effect on the RGC population (Table 1).

Next, we wondered whether minocycline treatment during these first days would suffice or, on the contrary, if we stopped the treatment RGCs would then die. Thus, animals were treated for 9 days, and analysed 9 days after the last dose. We considered 9 extra days because this was the time frame of RGC death without treatment. In addition, because in the contralateral retina RGC loss did not progress with time and to reduce the number of animals, we did not analyze the injured retinas as we would have to have had a group of ONC 2 mm without treatment and analyzed it at 18 days. As observed in Figure 6A,B and Table 1, suspension of minocycline treatment for 9 days did not affect RGCs rescue.

In agreement with these data, in all the treated groups Brn3a fluorescence signal per RGC was kept within intact levels (Figure 6C). Furthermore, the mRNA level of Pou4f1 was significantly higher in injured and contralateral retinas from the treated group (Figure 6D) compared to ONC alone.

### 2.7. Minocycline Treatment Reduces Microglial Activation and Increases the Expression of M2 Microglial Transcripts and of Anti-Inflammatory Mediators

**Figure 7:**
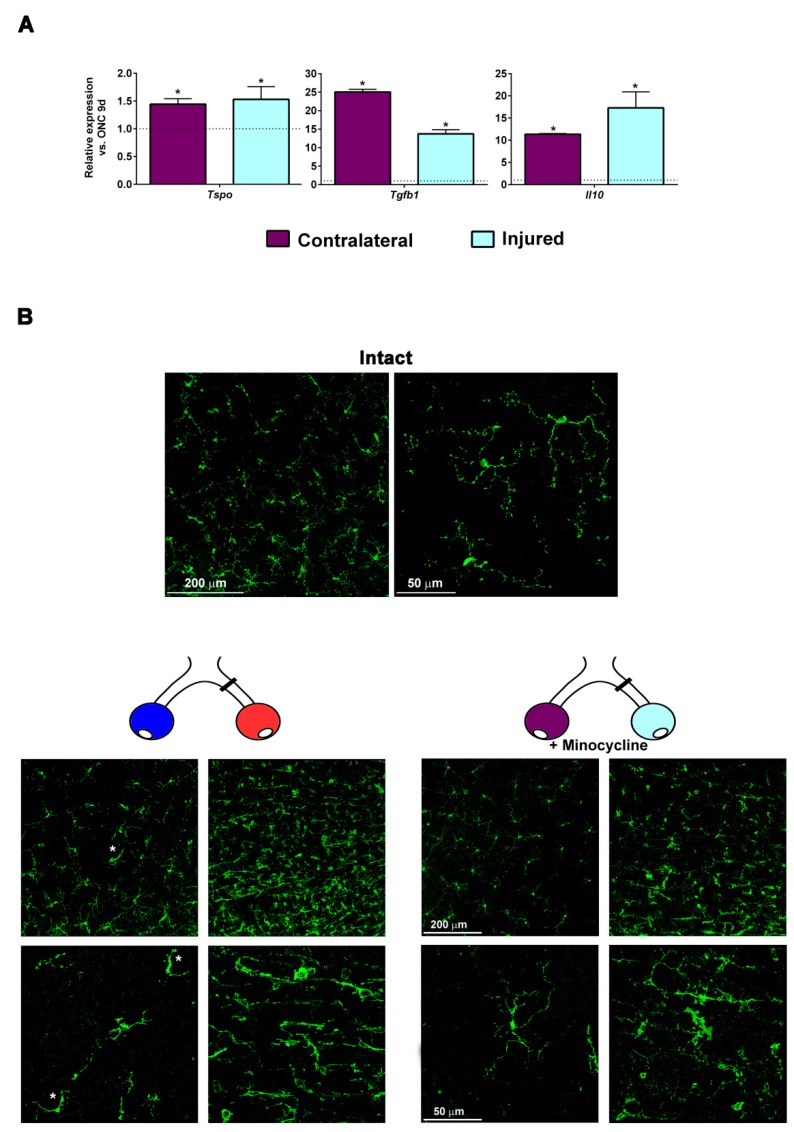
Minocycline treatment reduced microglial activation and increased the expression of M2 microglial transcripts and of anti-inflammatory mediators. (**A**) Bar graphs showing the expression level ± S.E.M. of *Tspo* (translocator protein 18kDa), *Tgfb1* (transforming growth factor β1) and *IL10* (interleukin 10) transcripts in the injured (ice blue) and contralateral (purple) retinas from the ONC + minocycline 9 day group compared to the ONC 9 day group (dotted line, value 1). * *p* < 0.05 compared to ONC 9 days *(t-*test). *n* = 4 retinas per group. (**B**) Confocal images from flat mounts showing Iba1^+^ microglial cells in intact, contralateral, and injured retinas analyzed 9 days after ONC 2 mm and treated or not treated with minocycline. In the contralateral retinas of untreated animals, some microglial cells showed signs of activation (asterisks) such as bipolar morphology and a bigger soma, an activation that was not observed in minocycline-treated animals. In the injured retinas, microglial activation looked similar in treated and untreated retinas, however minocycline treatment reduced the number of microglial cells.

In the same retinal extracts as above, we measured the regulation of translocator protein 18kDa (*Tspo*) a negative regulator of neuroinflammation and inducer of the expression of M2 microglial genes [34,35], and of two anti-inflammatory molecules, interleukin 10 (*IL-10*) and transforming growth factor β1 (*Tgfb1*). All of them were significantly up-regulated in both retinas from the minocycline-treated group compared to ONC alone (Figure 7A).

Finally, Figure 7B shows that minocycline treatment decreased the number of microglial cells as well as their activation, in agreement with previous reports [36,37,38].

## 3. Discussion

In this work, we showfor the first time that in pigmented mice the intraorbital distance at which the optic nerve is axotomized does not affect the course of RGC loss in the injured retina, but that it influences the kinetics of RGC loss in the contralateral un-injuredone. The loss of RGCs in the contralateral retina is diffuse, amounts for 15% of the original population, does not progress with time, and is completely prevented by microglial inhibition or by anti-inflammatory therapy. Thus, this bilateral response precludes the use of the un-injured retinas as controls of the RGC population.

The intraorbital access to the optic nerve is up to 2 or 3 mm from the optic disk in mice and rats, respectively. In rats, RGC death in the injured retina is quicker when the axotomy is inflicted at 0.5 than at 3 mm, and the difference is bigger at longer distances, which are reached by intracranial axotomy [9]. In mice, however, the course of RGC death is the same between the two most distant intraorbital locations. We do not know why, but it may be related with the rate of RGC loss, which is faster in mice (reviewed in [12]). At the practical level, this result means that anatomical data from reports inflicting axotomy at different intraorbital distances can be compared.

After the unilateral ONC, both retinas showed an early down-regulation of RGC-specific transcripts, which was caused mainly by a lower expression per RGC. Then, there was a rebound up-regulation that was transitory in the injured retinas because of the massive RGC death. However, the expression of Brn3a per surviving RGC recovered to normal levels during the second phase of RGC death (from 14 days onwards), indicating that after the initial shock, surviving RGCs gradually regained their physiological state. In the contralateral retina, the up-regulation of RGC markers was permanent, and with levels higher than in intact retinas, with some temporal differences among markers.

In previous studies from our group in which we analyzed the whole population of RGCs in albino mice long-term after axotomy [18], the number of RGCs in the un-injured retina was lower than in intact animals. This decrease was not significant, concurring with the fact that although there were RGCs degenerating across the retina they were few in number, and so RGC death was not detected [18]. Interestingly, Macharaza et al. [14] demonstrated that in albino rats a unilateral axotomy caused the death of ipsilateral RGCs in the contralateral retina, a death that was controlled by intravitreal administration of dexamethasone. The uncrossed projection in rodents is <4%, and it is further reduced in albino animals [29,39,40,41,42,43]. In our works in albino animals, we did not specifically identify these ipsilateral-RGCs but quantified the whole RGC population [1,18]. All in all, these data indicate that albino animals also have a bilateral response involving RGC death, only in that it is either mainly restricted to the ipsilateral RGCs or, most probably, the global effect on the retina is not big enough to reach significance.

Other groups have successfully prevented the contralateral response using anti-inflammatory [14] or immunosuppressive therapies [19]. Here, we treated the animals with an NSAID less toxic than dexamethasone [14], and instead of immunosupression [19] we chose to inhibit microglial cells with minocycline. These treatments rescued all RGCs in the contralateral but had no effect in the injured retinas. Meloxicam is a NSAID that acts through the inhibition of cyclooxygenase 2, the key enzyme for the production of inflammatory cytokines. Systemic treatment with meloxicam has been shown to inhibit the activation of microglial cells [28], and to be neuroprotectant after spinal cord injury [27,44] and ischemia reperfusion [44]. Minocycline is a tetracycline antibiotic that crosses the blood–brain/retinal barrier and exerts neuroprotection [26,45] through two main mechanisms: apoptosis inhibition (caspases 1 and 3) and suppression of microglial activation [23,24,25]. The lack of rescue in the injured retinas is in agreement with Hilla et al. [33] who demonstrated that in mice microglial cells are irrelevant for RGC death after optic nerve crush. Nevertheless, we did expect some positive effect with minocycline in the injured retinas because caspase 3 inhibition delays axotomy-induced RGC death [6]. The lack of neuroprotection with minocycline in the injured retinas couldbe due to the dose of the antibiotic, not enough to inhibit caspase 3 but enough to affect microglial cells, since our data show that in the minocycline group microglial cells were fewer and less activated, and anti-inflammatory mediators and markers of M2 microglial cells were up-regulated.

**Figure 8:**
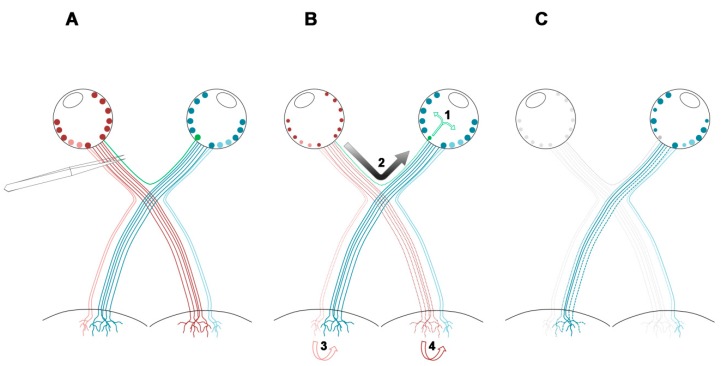
Mechanisms implicated in the contralateral response.(**A**) In rodents, ~96%–99% of the RGCs project to the contralateral superior colliculi (dark red, dark blue lines [51,52]). The ipsilateral projection (light red, light blue lines) amounts for 1.5% to 4% of the total RGCs. This uncrossed projection is significantly smaller in albino animals [29,39,40,41,42,43]. Finally, very few RGCs project to the other retina (green line, [46,47,48,49,50]). Upon optic nerve axotomy, the parent RGCs will die by the direct injury ((**B**) dotted lines, smaller RGC somas; (**C**) grey somas and axons). The massive loss of RGCs and the degeneration of their axons will cause a cascade of events ending in the death of RGCs in the un-injured retina. (**B1**) Stress/danger signals from the retino-retinal RGCs affected by the axotomy. (**B2**) Propagation of the glial reaction through the optic chiasm. This response would affect the optic nerve axons of the contralateral retina ((**C**) dashed blue lines), and eventually would reach the un-injured retina, impacting on the intraretinal axons and/or the RGC somas. (**B3**) Reaction to the loss of the injured ipsilateral projection that damages the contralateral projection of the un-injured retina. (**B4**) Reaction to the loss of the contralateral projection that harms the ipsilateral projection of the un-injured retina [14]. The deafferentation of the superior colliculi would cause the retrograde death of the parent RGCs ((**C**) dotted blue lines). These mechanisms alone or in combination, together with a possible systemic response, would finally cause the death of RGCs in the un-injured retina ((**C**) smaller blue somas).

What are the causes of the contralateral response? Different non-exclusive mechanisms have been proposed, (summarized in Figure 8 and reviewed in [22]): (i) a direct response to the death of the retino-retinal projecting RGCs, although their death would not amount to the total loss reported here because the number of retino-retinal RGCs is very small in adult rodents (adult rats from 0 to 4; adult mice from 3 to 12) [46,47,48,49,50]; (ii) stress signals released from the injured retino-retinal RGCs that would activate glial cells, which in turn would trigger an inflammatory neurotoxic response; (iii) an inflammatory signal that reaches the contralateral retina but that is independent of the retino-retinal projection, such as propagation of the glial reaction in the damaged retina to the contralateral retina through the optic chiasm; (iv) a reaction to the deafferentation of the ipsilateral superior colliculi [14]; and finally, (v) some signals may travel systemically [21].

Because the loss of RGCs in the contralateral retina was observed earlier when the lesion was performed at 2 mm (closer to the contralateral site), it is plausible to think that the signal travels through the contralateral optic nerve, either from the damaged retina or from the deafferentated retinorecipient areas in the brain or both.

In albino animals, as mentioned above, the loss of RGCs in the contralateral retina is not significant. The main difference with pigmented animals is the ipsilateral projection. Thus, it follows that the deafferentation of the superior colliculi does have a negative impact on the still viable projections, an impact that would be more pronounced in species with a bigger ipsilaterally, such as primates or humans (Figure 8).

The contralateral death is stopped by anti-inflammatory treatment and so the starting signal must be pro-inflammatory engaging microglial cells which, in turn, may be the executors because their direct inhibition saves RGCs. Furthermore, the signal must occur once and early after the lesion because RGC death does not progress beyond 15%, and an early microglial inhibition seems enough to stop the degeneration even after suspension of the treatment.

Finally, the topography of RGC loss is diffuse across the retina, suggesting that whichever the origin of the triggering signal, it spreads throughout the whole contralateral retina.

In conclusion, we demonstrate here that a localized axonal injury to the CNS causes not only the death of the parent neurons, but also triggers a contralateral response that kills distant ones. The damage to the un-injured retina is controlled by anti-inflammatory and microglial inhibitory therapies and thus these treatments may be used as conjunctive therapy in unilateral retinal diseases or after traumatic brain injury affecting the optic nerve at the optic foramen. Finally, whether this effect is limited to the visual system or to bilateral projections in the CNS is not known, and further studies should be carried out.

## 4. Materials and Methods

### 4.1. Animal Handling

Adult pigmented C57Bl/6 male mice (30 g) were obtained from the University of Murcia breeding colony. All animals were treated in compliance with the European Union guidelines for Animal Care and Use for Scientific Purpose (Directive 2010/63/EU) and the guidelines from the Association for Research in Vision and Ophthalmology (ARVO) Statement for the Use of Animals in Ophthalmic and Vision Research. All procedures were approved by the Ethical and Animal Studies Committee of the University of Murcia, Spain (Approval number: A1320140704; aproved the 24 July 2014, extended 28 April 2016).

Animals undergoing surgery were anesthetized by intraperitoneal injection of a mixture of ketamine (60 mg/kg; ketolar, Pfizer, Alcobendas, Madrid, Spain) and xylazine (10 mg/kg; Rompum, Bayer, Kiel, Germany). Analgesia was provided by subcutaneous administration of buprenorphine (0.1 mg/kg; Buprex, Buprenorphine 0.3 mg/mL; Schering-Plough, Madrid, Spain). During and after surgery, the eyes were covered with an ointment (Tobrex; Alcon S.A., Barcelona, Spain) to prevent corneal desiccation. Animals were sacrificed with an intraperitoneal injection of an overdose of sodium pentobarbital (Dolethal, Vetoquinol; Especialidades Veterinarias, S.A., Alcobendas, Madrid, Spain).

### 4.2. Animal Groups and Experimental Design

(1) Intact, no surgery. (2) Toxicity: meloxicam or minocycline treatment in animals that did not undergo optic nerve lesion. (3) Optic nerve crush (ONC) at 0.5 mm from the optic disc. (4) ONC at 2 mm from the optic disc. (5) ONC at 2 mm from the optic disc + minocyclin treatment. (6) ONC at 2 mm from the optic disc + meloxicam treatment.

**Figure 9:**
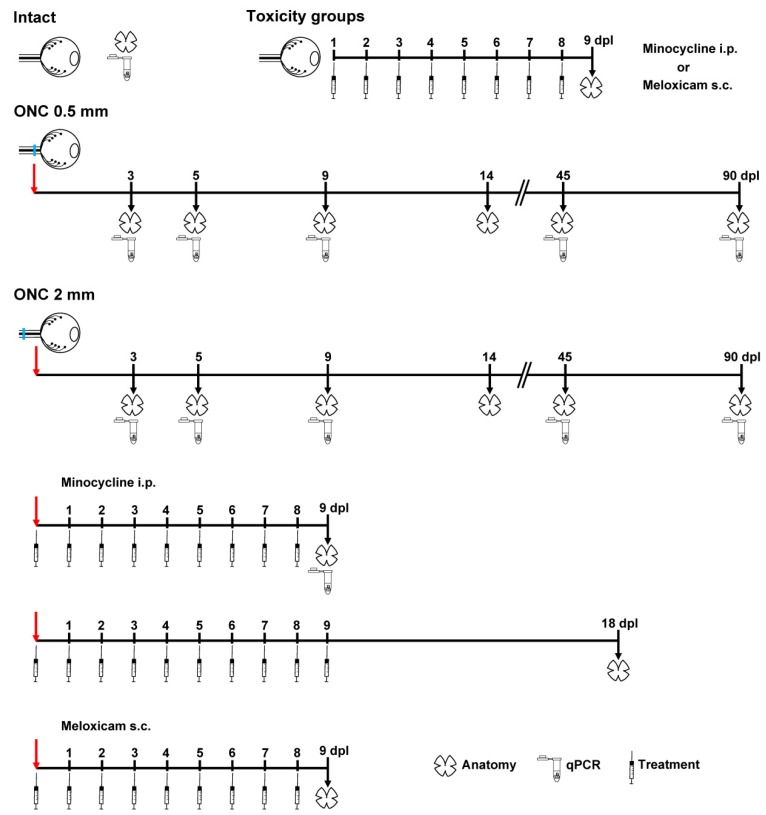
Experimental design: animal groups, timeline, and analyses. Toxicity analyses were done on animals that did not undergo optic nerve lesion. Both retinas of each animal were analyzed except for the ONC + meloxicam group at 18 days of which only the contralateral retinas were studied. Red arrows mark the surgery and black arrows the days of analyses. Retinal outlines indicate anatomical analyses: RGC quantification in all groups, and microglial morphology in intact, ONC 2 mm, and ONC 2 mm + minocycline at 9 dpl. Microtubes mean qPCR. Syringes mark the intraperitoneal (i.p.) or subcutaneous (s.c.) treatment with minocycline or meloxicam, respectively. The number of retinas analyzed per group, time point, and technique ranged from 4 to 10 (more details in figure legends). dpl: days post-lesion.

For experimental design, see Figure 9.

Both retinas of each animal were analyzed, the left retinas (injured), and the contralateral to the lesion, right un-injured retinas (henceforward contralateral retinas). The number of retinas per group and time point was 4–10 for anatomical analysis (see results for a detailed *n*), and 4 for qPCR.

### 4.3. Optic Nerve Crush

The left optic nerve was crushed at 0.5 or 2 mm from the optic disc following previously described methods [6]. We chose 2 mm because it is the further the optic nerve in mice that can be safely accessed intraorbitally. Briefly, to access the optic nerve at the back of the eye, an incision was made in the skin overlying the superior orbital rim, the supero-external orbital contents were dissected, and the superior and external rectus muscles were sectioned. Then, the optic nerve was crushed at 0.5 or 2 mm from the optic disc for 10 s using watchmaker′s forceps. Before and after the procedure, the eye fundus was observed through the operating microscope to assess the integrity of the retinal blood flow.

### 4.4. Minocycline and Meloxicam Treatment

Right after the ONC, animals received an intraperitoneal injection of minocycline (45 mg/kg; Sigma Aldrich, Madrid, Spain) or a subcutaneous injection of meloxicam (2 mg/kg, Meloxidolor; 5mg/mL, FatroIberica, Spain). Thereafter, they were treated daily for 9 days (Figure 9).

### 4.5. Immunodetection

Animals were perfused transcardially with 0.9% saline solution followed by 4% paraformaldehyde in 0.1 M phosphate buffer. Retinas were dissected as flattened whole-mounts as previously described [6].

Immunodetection was carried out as previously reported [4]. RGCs were detected with mouse α-Brn3a (brain-specific homeobox/POU domain protein 3A; 1:500; MAB1585, Merck Millipore; Madrid, Spain) and microglial cells, with rabbit α-Iba1 (ionized calcium-binding adapter molecule 1; 1:500; ab178846, Abcam, Cambridge, United Kingdom). Secondary detection was carried out with donkey α-mouse IgG1-Alexa fluor 594 and donkey α-rabbit Alexa 488 (1:500; Molecular Probes; Thermo Fisher Scientific, Madrid, Spain).

### 4.6. Image Acquisition and Analysis

Brn3a images were acquired using an epifluorescence microscope (Axioscop 2 Plus; Zeiss Mikroskopie, Jena, Germany) equipped with a computer-driven motorized stage (ProScan H128 Series; Prior Scientific Instruments, Cambridge, United Kingdom) controlled by image analysis software (Image-Pro Plus, IPP 5.1 for Windows; Media Cybernetics, Silver Spring, MD). Retinal photomontages were reconstructed from 154 (11 × 14) individual images [4]. Microglial magnifications were acquired with a confocal microscope (Servicio de Apoyo a la Investigación, UMU/IMIB-Arrixaca, Leica SP8; Leica Microsytems, Wetzlar, Germany).

The whole population of Brn3a + RGCs was quantified automatically and their distribution assessed by neighbour maps using previously reported methods [53]. These maps depict, using a colour scale, the number of neighbours around a given cell in a radius of 0.200 mm. All maps were plotted using SigmaPlot (SigmaPlot 9.0 for Windows; Systat Software, Inc., Richmond, CA, USA).

Brn3a expression intensity was measured in images acquired with the same settings, as reported [29], with the exception that the graphs were plotted using GraphPad Prism v.7.

### 4.7. Quantitative Real Time PCR

Retinas were freshly dissected and immediately frozen on dry ice (*n* = 4 per group and time point). Total RNA was extracted using Trizol reagent (Thermo Fisher Scientific) and the RNA samples were dissolved in 20 µL Milli-Q water. Total RNA concentration was determined using SimpliNano (GE Healthcare Life Sciences, Madrid, Spain). Complementary DNA amplification was performed according to the instructions provided by the manufacturer SuperScript IV VILO Master Mix, Thermo Fisher Scientific) using 1 µg total RNA.

Mouse predesigned SYBR green primers (pair 1) for *Rbpms* (RNA-binding protein with multiple splicing), *Sncg* (Synuclein Gamma), *Casp3* (Caspase 3), *Tspo* (translocator protein 18kDa*), IL10* (interleukin 10), *Tgfb1* (transforming growth factor β1), *Actb* (Actin Beta), and *Hrpt* (Hypoxanthine-guanine phosphoribosyltransferase) were purchased from Sigma Aldrich. *Gapdh* (Glyceraldehyde 3-phosphate dehydrogenase) primers (Mm_Gapdh_3_SG QuantiTect Primer Assay) were from Qiagen (Barcelona, Spain). *Pou4f1* (POU domain, class 4, transcription factor 1) primers were custom made (*Pou4f1* Fw 5′AGAAGCTTTTAAGCCACTTCC3′ Rev 5′TAGAACAGCACATTCAAACC3′; Sigma Aldrich).

SYBR Premix Ex Taq II (Tli RNaseH Plus, TaKara; Thermo Fisher Scientific)-based qPCR was carried out by the Genomic Platform at the IMIB-Arrixaca in a final volume of 5 μL with a primer concentration of 450 nM using the QuantStudio 5 (Applied Biosystems; Thermo Fisher Scientific). Technical triplicates were done for each sample.

The Ct values were converted to relative quantification using the 2^ΔΔCt^ method [54]. Three candidate housekeeping genes (*Hprt, Gadph,* and *Actb*) were evaluated using NormFinder [55]. The most thermo stable was *Hprt*, and thus its Ct was used as housekeeping value.

### 4.8. Statistics

Data were analyzed and plotted with GraphPad Prism v.7 (GraphPad San Diego, CA, USA). Tests are detailed in the Results section. Differences were considered significant when *p* < 0.05. Data are presented as mean ± standard deviation (S.D.) or mean ± standard error of the mean (S.E.M.).

## Figures and Tables

**Table 1:** Total number of RGCs. Mean number ± standard deviation (SD) of the total population of RGCs quantified in the injured and contralateral retinas from each group and time point. In the injured retinas, RGC death was first significant at day 3 after either lesion. In the contralateral un-injured retinas, RGC death was significant at day 9 in the ONC 2 mm group, and at day 45 in the ONC 0.5 mm group. Minocycline or meloxicam treatment for 9 days did not affect the population of RGCs in non-injured animals (toxicity assays), but rescued RGCs in the contralateral retinas (ONC 2 mm + Mino (minocycline), ONC 2 mm + Melox (meloxicam)). This rescue was maintained 9 days after ending minocycline administration (ONC 2mm + Mino + 9d). In the injured retinas, neither treatment had an effect on RGC survival.

					Time Post-Lesion or Treatment					
Group	Retina		Intact	3d	5d	9d	14d	18d	45d	90d
Intact		Mean	44,646							
		SD	695							
Toxicity		Mean				43,364				
Minocycline		SD				1926				
Toxicity		Mean				42,896				
Meloxican		SD				1593				
ONC 0.5 mm	Contralateral	Mean		41,057	41,525	42,151	40,989		36,349 ***	37,800 ***
		SD		1436	2022	1652	1137		1971	1874
	Injured	Mean		37,178 *	22,910	8444	4022		1494	687
		SD		2561	2779	402	269		295	304
ONC 2 mm	Contralateral	Mean		40,939	41,005	36,117 ****	36,349 ***		36,877 ***	38,262 **
		SD		1546	2257	1425	1971		1772	1366
	Injured	Mean		37,120 *	23,285	9759	5128		1209	797
		SD		1501	1696	2385	1891		236	224
ONC 2 mm + Mino	Contralateral	Mean				43,888 ^†††^				
		SD				1919				
	Injured	Mean				10,854				
		SD				722				
ONC 2 mm + Mino + 9d	Contralateral	Mean						41,478 ^†^		
		SD						1299		
ONC 2 mm + Melox	Contralateral	Mean				42,423 ^†^				
		SD				918				
	Injured	Mean				9901				
		SD				1252				

* Statistically significant compared to intact retinas (**** *p* < 0.0001, *** *p* < 0.001, ** *p* < 0.01, * *p* < 0.05, one-way ANOVA, Kruskal–Wallis, Dunn′s multiple comparisons tests) ^†^ Statistically significant compared to contralateral retinas from the ONC 2 mm group analyzed at 9 days (^†††^ *p* < 0.001, ^†^ *p* < 0.05, one-way ANOVA, Kruskal–Wallis, Dunn’s multiple comparisons tests).

## Abbreviations

CNS	Central nervous system
NSAID	Non-steroidal anti-inflammatory drug
ONC	Optic nerve crush
RGC	Retinal ganglion cell
